# EVALUATING VIRTUAL MONTESSORI DEMENTIA TRAINING USING NORMALIZATION PROCESS THEORY AMONG VA NURSING HOME STAFF

**DOI:** 10.1093/geroni/igae098.2697

**Published:** 2024-12-31

**Authors:** Katherine Kennedy, A Snow, Whitney Mills, Amy Mochel, Sylvia Haigh, Teddy Bishop, Christine Hartmann, Michelle Hilgeman

**Affiliations:** VA Providence Healthcare System, Center of Innovation in Long-Term Services and Supports, Providence, Rhode Island, United States; Tuscaloosa VA Medical Center, Tuscaloosa, Alabama, United States; VA Providence Health Care System, VA Providence Health Care System, Rhode Island, United States; Providence VA Healthcare System, Providence, Rhode Island, United States; Providence VA Medical Center, Providence, Rhode Island, United States; Tuscaloosa VA Medical Center, Tuscaloosa, Alabama, United States; VA Bedford Healthcare System & University of Massachusetts Lowell, Bedford, Massachusetts, United States; Tuscaloosa VA Medical Center, Tuscaloosa, Alabama, United States

## Abstract

This paper uses Normalization Process Theory (NPT) to examine staff impressions of Montessori-based program training and implementation at Veterans Affairs Community Living Centers (VA CLCs; nursing homes). We conducted a mixed-methods evaluation of Montessori Approaches to Person-Centered Care (MAP-VA) at eight VA CLCs. Trainings were conducted as either a live virtual course or a pre-recorded asynchronous class. Two NPT constructs, coherence building and cognitive participation, informed qualitative interview questions, surveys, and analyses focused on staff movement from knowledge to action during initial implementation. Data collection included staff-completed standardized post-training exams (N=906), post-training evaluations (N=761), and optional validated surveys on perceptions of Montessori training (N=307). Champions (peer-leaders) from each CLC completed semi-structured qualitative interviews post-training (N=22). The majority of staff (83%-90%) passed all courses. Staff evaluated the training highly (80%+ agreement) on learning relevant new knowledge and confidence applying new skills. On average, staff felt MAP-VA would become a normal part of their work (7.68/10 scale) and reported increased familiarity with Montessori approaches after training (p =.002). Qualitative interview data from staff trained in Montessori supported three themes concordant with the NPT dimensions of coherence building and cognitive participation. Montessori virtual training resulted in high levels of coherence and cognitive participation among multidisciplinary staff, evidenced by high knowledge, self-efficacy, and readiness to act. The asynchronous and synchronous trainings were accessible, relevant, and supported diverse learners. Virtual dementia training can be an effective and efficient method of teaching nursing home staff about Montessori approaches to reduce responsive behaviors.

